# Precise Coulometric Titration of Acids and Bases

**DOI:** 10.6028/jres.063A.008

**Published:** 1959-10-01

**Authors:** John K. Taylor, Stanley W. Smith

## Abstract

Apparatus and techniques are described for the titration of acids and bases by constant-current coulometry. The precision and accuracy of the method are indicated by titrations of benzoic acid, potassium acid phthalate, adipic acid, hydrochloric acid, and sodium carbonate. Standard deviations of ±0.003 percent have been obtained which indicate that the reliability of the method is equal to or exceeds that of classical analytical procedures.

## 1. Introduction

In coulometric analysis, the laws of electrolysis are applied to analytical chemistry. The material to be analyzed is placed in an electrolytic cell and is neutralized, oxidized, reduced, or precipitated, as the case may be, by a measured quantity of electricity. From this measurement, Faraday’s laws are used to determine the amount of chemical substance originally present.

While this technique has been used only for the past 20 years, and particularly in the past decade, numerous procedures have been developed, especially in microchemical analysis, where coulometry has definite experimental advantages over conventional volumetric methods [1, 2, 3].^2^

With the evaluation of the faraday constant with ever-increasing precision, by purely physical methods, coulometry offers the attractive possibility of permitting chemical standardizations to be made directly with reference to fundamental electrical constants. In fact, a proposal has been made to the International Union of Pure and Applied Chemistry to accept the electron as a fundamental chemical standard [4].

The National Bureau of Standards has had a continuing interest in developing analytical methods of the highest accuracy for the determination of the purity of materials suitable for chemical standards. Because they differ so much from conventional procedures, the precision and accuracy of coulometric methods are being extensively investigated. A preliminary report on this program was presented to the National Academy of Sciences at its meeting in Washington in November 1956 [5]. The present paper is a detailed account of the apparatus and techniques developed for coulometric titrations of high precision. It is shown that the reliability of the results obtained is equal to or exceeds that of the most careful determinations made by classical analytical methods.

## 2. General Considerations

### 2.1. Classification of Methods

Coulometric titrations may be classified according to whether the electrode process directly or indirectly influences the composition of the solution. Acidimetric titrations in which hydrogen ion is reduced at the cathode are examples of the former type, while the precipitation of halide ion by coulometrically generated silver ion is an example of the latter type.

A further classification may be made on the basis of the type of current control. Controlled-potential coulometry consists in maintaining the electrode potential at a selected value that produces only a desired electrode reaction. In this case, the current decreases continuously and exponentially during the electrolysis, and asymptotically approaches zero or some small constant value. The current is integrated during the course of the electrolysis, and is discontinued when it does not differ significantly from this terminal value.

Constant-current coulometry is based on the maintenance of an accurately measured current and the determination of the time required to complete the desired reaction. Integration is not required, but the electrolysis must be discontinued at the end point of the desired reaction. End-point determination may be made by conventional methods such as potentiometric, colorimetric, amperometric, or thermometric techniques, depending on the particular reaction.

### 2.2. Advantages of Coulometric Titrations

In addition to the possibility of making determinations based on physical standards, coulometric measurements have several obvious advantages. Electrochemical reactions may be considered as titrations with electrons, a universal “reagent.” This reagent may be added uniformly at any desired rate by methods more convenient than those required to dispense conventional chemical substances. Uncertainties in composition of standard solutions are eliminated as are volumetric errors. The high precision with which electrical measurements can be made is a distinct advantage in coulometric methods. Methods for the control of electrical circuits are well-developed, so that coulometric titrations are especially adaptable for automatic operation.

### 2.3. Sources of Error

It is of prime importance that the stoichiometry of the electrode process and any of its indirect reactions be completely defined. Any uncertainty in the current-chemical reaction relationship will result in a direct error in the determination. In general, reactions which proceed with less than 100-percent current efficiency are not useful.

In many cases, the reverse of the desired reaction occurs at the second electrode, or the product of the reaction at this electrode may interfere with the reaction of primary interest. Accordingly, it is frequently necessary to isolate the second electrode in a separate compartment that is in electrolytic contact with the electrolysis chamber. In such cases, it is necessary to minimize losses due to diffusion or to bulk transfer of the solution. A further source of error may arise from ionic migration from the electrolysis chamber. The familiar technique of addition of a supporting electrolyte will minimize this error.

Errors, both accidental and systematic, can arise in the determination of the end point as in conventional titrations. Errors can arise from faulty calibration of the apparatus used for the measurement of the current and the time.

## 3. Apparatus and Procedure

### 3.1. Apparatus

The acidimetric titrations to be described were made by the method of constant-current coulometry. The cell consists of a platinum cathode and a silver anode, with an alkali chloride solution as the supporting electrolyte. The cell reaction results in the reduction of one equivalent of hydrogen ion (liberation of one equivalent of hydrogen gas) at the cathode, and the formation of one equivalent of silver chloride at the anode for each faraday of electricity.

**Figure 1:**
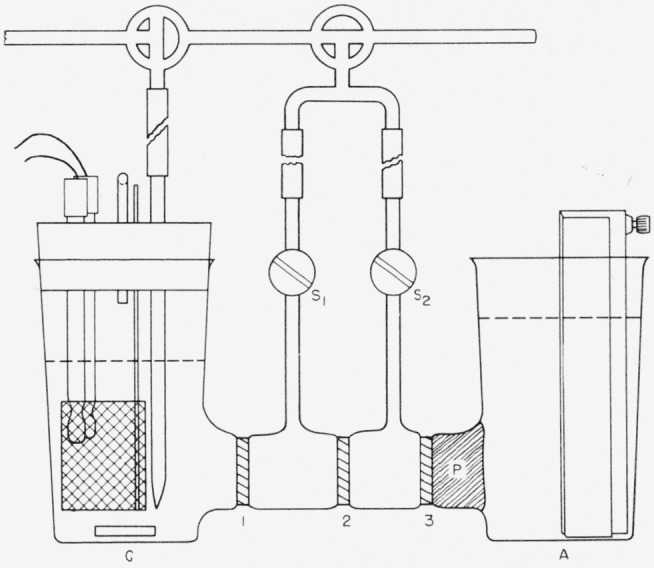
Coulometric titration cell.

Because of the small but finite solubility of silver chloride in the electrolyte, a small amount of silver is reduced and deposited on the cathode when a single compartment cell is used. To overcome this source of error, the titration cell shown in figure 1 was designed. The cathode chamber, *C*, contains the platinum-gauze cathode and also the glass and calomel electrodes to indicate the end point *p*H of the solution. An encapsulated magnet bar is used to stir the solution. A tube is provided to admit nitrogen to the cell to remove carbon dioxide. The compartment is sealed with a rubber stopper and a bubble trap to exclude air.

The anode compartment, *A*, contains a silver anode of large area so that the anode reaction results only in the precipitation of silver chloride on its surface, no acid being formed. It is fabricated from a sheet of silver 7.6 by 15 by 0.065 cm in thickness. This is corrugated and placed as far as possible from the connecting port, to assure large current capacity and good current distribution. Such an electrode has a capacity of more than 1,500 coulombs and can be regenerated by electrolytic reduction of the silver chloride. More conveniently, however, the electrode may be renewed by chemical solution of the chloride in ammonia.

The actual compartments are 400-ml beakers of the electrolytic type, without lip. They are connected by a 35-mm tube, approximately 125 mm in length, in which are sealed sintered glass disks as shown in the figure. Sintered disks 1 and 2 are of medium porosity while 3 is of fine porosity. For convenience, this bridge is made from commercially available “sealing tubes.” Each of the center compartments has a tube attached to permit emptying and filling under nitrogen pressure as will be described. A plug of agar gel, *P* (3 percent agar-agar in 1 *N* potassium chloride) is placed at the anode side of disk 3 to prevent liquid transfer into or from this compartment.

**Figure 2:**
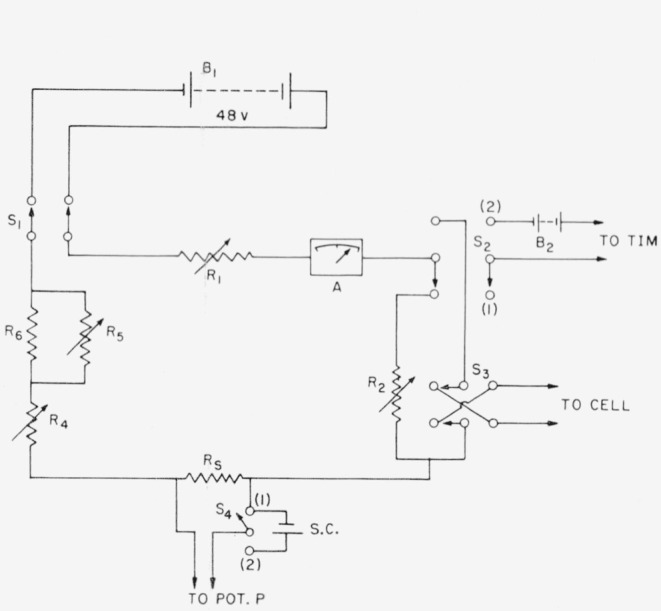
Electrical circuit diagram for coulometric titrations.

The circuit used for the maintenance of the constant current is shown in figure 2. A storage battery, *B*_1_ of 48 v serves as the current source. It permits a high-resistance circuit so that small changes in cell resistance (voltage drop across the cell) during the course of the electrolysis will have a proportionally smaller effect on the current. A voltage dropping resistor, *R*_1_, is immersed in an oil bath to aid in heat dissipation and stabilize its resistance during a measurement. The combination, *R*_5_*R*_6_, consists of a fixed resistance and a carbon-compression resistor in parallel. It has an overall variation of about 20 ohms and is adjusted continuously during the measurement to maintain the current constant. Resistance *R*_4_ is used for coarse adjustment of the current.

A variable resistor, *R*_2_, is adjusted to have, within narrow limits, the same resistance as the cell. The switch *S*_2_ when in position (1), permits the current to flow through this “dummy” resistor for preliminary adjustment. Ammeter *A* is used to facilitate the initial adjustment of the current. Switch *S*_2_, when thrown to position (2), causes the current to flow through the cell and simultaneously triggers the timing device.

The current is measured by the voltage drop across the standard resistor, *R_s_*, a 5- or a 10-ohm precision resistor, depending on the value of the current. These resistors are placed in a constant-temperature oil bath. Saturated standard cells, suitably thermostated, serve as the primary reference of voltage. The voltage drop across *R_s_* is compared with the voltage of the standard cell, any difference being measured by potentiometer *P.*

During a titration, the current is maintained constant by manually adjusting it in the following manner. By preliminary trials, it is possible to adjust the value of the dummy resistor to approximately that of the cell. Accordingly, when the switch is thrown to the “electrolysis” position, potentiometer *P* can be adjusted quickly to correspond to the cell current. The potentiometer key is then locked in the closed position. Any change in the current causes the galvanometer to deflect from the null position, and this is immediately corrected by adjustment of resistor *R*_5_.

Initially, the time interval was determined by means of an electric clock, driven by a tuning-fork regulated constant-frequency device, capable of a precision of one part in a hundred thousand. To secure higher precision in timing, and to eliminate errors due to clutch slippage in the clock, a quartz crystal-controlled time-interval meter (TIM) was used in later experiments. The meter is compared occasionally with NBS standard time signals to check its performance. It is capable of an accuracy of one part in a million, and has never differed from the time signal by this amount.

The *p*H measurements, on which the end point determination is based, are made with conventional glass electrodes and a line-operated indicator. A suitable resistance is connected in series with the meter of the instrument and the voltage drop across this is measured (14.5 mv per *p*H unit). This effectively expands the scale of the instrument since this voltage can be readily measured to a few hundredths of a millivolt with an ordinary potentiometer. The stability of the *p*H meter during the period of a determination of the end point is sufficient to warrant this procedure.

All apparatus for making the electrical measurements, including standard cells, standard resistors, and potentiometers, was calibrated by the electrical division of this Bureau. The value for the faraday constant used was 96,495.6 coulombs [7].

### 3.2. General Procedure

A 1 *N* solution of potassium chloride is added to the anode compartment of the cell. A weighed portion of solid potassum chloride is placed in the cathode compartment and, after flushing with nitrogen, freshly boiled distilled water is added in sufficient quantity to make the resulting solution 1 *N.* Pure nitrogen is then introduced through the connecting tube and into the solution to remove dissolved carbon dioxide. To insure that the electrolyte is sufficiently acid to facilitate this operation, a few drops of acid are intentionally added to bring the electrolyte to a *p*H of 5.

After purging is complete, the added acid is neutralized as follows. Stopcocks *S*_1_ and *S*_2_ are opened to the atomsphere to permit the solution to flow into the connecting tube to a depth of about 2 mm. A current of 10 ma is passed until the glass electrode indicates a *p*H value of 7.0.^3^ Nitrogen is then admitted into the side tube to “blow” the contents back into the cathode compartment. This procedure is repeated until no further change in *p*H results on mixing and the *p*H remains at 7.00.

By appropriate manipulation of the stopcocks, the connecting tube is then filled with neutralized catholyte. The stopper is momentarily removed to introduce the acid to be titrated.

The current is then adjusted to a value of 200 ma and the solution is electrolyzed until the glass electrode indicates a value very close to the end point. At this rate of electrolysis, the acidity of the electrolyte in the vicinity of the end point changes more rapidly than the glass electrode can respond. Accordingly, some experience may be required to insure that the end point is not overrun. Nitrogen pressure is then used to push the contents of the connecting tube into the cathode compartment for mixing.

The connecting tube is then filled with electrolyte to a depth of 2 mm, as previously described, and the solution is electrolyzed at a current of 10 ma to within about 0.5 *p*H unit of the end point. At this current, the glass electrode follows the change of *p*H very closely so that there is little danger of overrunning the desired value.

Electrolysis is then carried out, stepwise, for convenient time intervals and the *p*H is recorded for each interval. From these data, a conventional differential potentiometric plot is constructed to determine the end point. The connecting tube is then flushed several times with catholyte until no further change in *p*H results. From the change in *p*H resulting from this flushing, and the titration curve, a small correction is made to the end-point time already determined.

**Figure 3:**
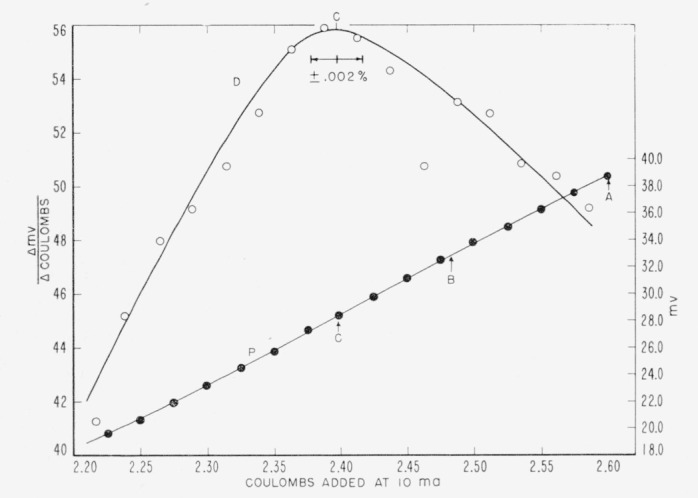
Typical end-point determination.

A typical end-point determination is shown in figure 3. Curve *P* represents the plot of the potential of the glass electrode indicator as a function of the elapsed time. Curve *D* is the differential plot used to locate the end point, identified as point *C.* The tolerance limits for the location of the end point that would correspond to an error of ±0.002 percent in the titration are indicated. Point *A* represents the time at which electrolysis was discontinued, while point *B* indicates the final *p*H value for the solution, after rinsing was completed. The corrected end point is then time *C*+(time *A*—time *B*).

## 4. Titration of Potassium Hydrogen Phthalate

### 4.1. Procedure

The potassium hydrogen phthlate used in these experiments was NBS Standard Sample 84d. Small amounts (15 to 20 g) were crushed in a mortar and dried at 120° C for 2 to 3 hr. The samples were stored in a desiccator until used.

After removal of carbon dioxide from, and neutralization of the supporting electrolyte, the rubber stopper was removed momentarily, and the acid (2 g), weighed to 0.01 mg, was dumped into the cell from a weighed platinum boat. Reweighing of the boat gave the amount of acid introduced into the cell.

The acid was then electrolyzed at a current rate of 200 ma and finally at a rate of 10 ma, as described above.

### 4.2. Results

The results obtained for a number of titrations of standard sample 84d are given in columns 2 and 3 of table 1. The values given represent the purity found for the sample on the basis of an equivalent weight of 204.216. The electric clock was used for measurement of time in the case of the first group while the time-interval meter was used in the second series.

Although separated by a period of time of about 1 year, and determined by slightly different techniques, the agreement of the averages for the two series is excellent and shows the high precision of coulometric titrations.

On the basis of very careful titrations by conventional methods, sample 84d was indicated originally as having an assay of 100.04 percent. Subsequently, Bates and Wichers [6] compared this material with specially purified benzoic acid by differential titration and reported an assay of 99.987 percent (*σ* = 0.002). The agreement of the values of table 1 with this latter value is confirmation that the coulometric method is one of high accuracy.

## 5. Titration of Benzoic Acid

### 5.1. Procedure

The benzoic acid used was prepared at NBS for use as a calorimetric standard. The acid was a coarse, crystalline material and has been shown by other investigators [5] not to take up water under conditions prevailing in this locality. Accordingly, it was used without further purification or drying.

After preparation of the cell as described previously, a 1-g sample was weighed in a platinum boat and transferred to the cathode compartment. A period of about 30 min was allowed before starting the electrolysis, to permit a substantial amount of the acid to dissolve. Additional acid dissolves during the initial reduction period. When the reaction is approximately 98 percent complete, the current is discontinued, the connecting tube is blown dry, and sufficient time is allowed for the solution of the remainder of the sample (about 20 to 30 min). The titration is then completed with a current of 10 ma as already described. A final rinsing of the cell walls and stopper is accomplished with 15 percent, carbon dioxide free ethanol, to insure solution of residual traces of the acid.

### 5.2. Results

The results obtained in two independent series of measurements with different timing techniques are shown in columns 4 and 5 of table 1. The values reported represent the assay of the acid on the basis of an equivalent weight of 122.112.

By the differential method already referred to, Bates and Wichers [6] found the assay of this acid to be 99.996 percent (*σ* =0.001).

## 6. Titration of Hydrochloric Acid

### 6.1. Procedure

A solution of hydrochloric acid was prepared by weight dilution of constant-boiling acid. Based on the pressure during the distillation of the starting material and the dilution data, the acid was calculated to have a concentration of 0.11485_2_ milli-equivalents per gram of solution [8].

After the usual preparation of the cell, 50 g of the acid was added from, a weight buret. Electrolysis was started immediately and continued to about 99.8 percent of completion. The connecting tube was then rinsed and the neutralization was completed at the 10-ma rate as described previously. In this case, it was not necessary to prepare an end point curve, due to the rapid change in *p*H at the end point. Thus, a difference in *p*H of 0.1 unit at the end point corresponded to 0.005 coulombs (0.5 sec at the 10-ma rate). It was only necessary to attain the same end point obtained in the preliminary conditioning of the cell, a value easily determined within 0.02 *p*H units.

### 6.2. Results

The results of seven coulometric titrations of this acid are given in column 6 of table 1. The reported value is the “purity” of the acid on the basis of its calculated concentration from pressure-distillation data. Expressed in other terms, the solution was found to have a concentration of 0.114850 milli-equivalents per gram, as compared with the calculated value of 0.11485_2_ indicated in section 6.1.

While the agreement with the calculated value is satisfactory, the precision of the determination is disappointing. On the basis of uncertainty of the end point, a reproducibility of at least 1 part in 100,000 would be expected. Undoubtedly, some of the error is that inherent in the dispensing of liquid samples. In order to estimate the magnitude of such errors, a series of measurements were made in which 50-g samples of water were dispensed from a weight buret into tared weighing bottles. The difference between the weight of water dispensed and the contents of the weighing bottles showed a variation of 3.6 parts in 100,000. If all of this were reflected in the uncertainty of the weight of the sample, the larger average deviation found in these measurements would be partially explained.

## 7. Titration of Adipic Acid

### 7.1. Procedure

The adipic acid used was reagent grade material which had been further purified by filtration and crystallization from the melt by Delmo Enagonio of the Bureau. The material was estimated to have an assay of 99.9+ percent. The titration procedure used was the same as that followed in the case of potassium acid phthalate, and the results are reported merely to show the adaptability of the method to a variety of acids.

### 7.2. Results

The results of a series of titrations to determine the purity of this sample of adipic acid are given in column 7 of table 1. The material was further recrystallized and the purity was found to be higher, as recorded in column 8. The smaller value for the standard deviation indicated that the homogeneity of the sample was also improved by the second purification.

## 8. Titration of Sodium Carbonate

### 8.1. Procedure

These experiments were performed to extend the method to the titration of bases. The sodium carbonate was reagent grade material which had been heated in a platinum dish for several hours at a temperature of 350° to 400° C. The assay was not determined by an independent method.

The electrolyte used was 1*−M* sodium sulfate, since chlorides would be oxidized at the anode if present in the solution. The agar plug was also made with 1*−M* sodium sulfate.

The solution was preconditioned as described previously. After addition of the base, the platinum electrode was made the anode and electrolysis was continued until approximately 99 percent of the base had been neutralized. The titration was completed at the 10-ma rate as before. Sufficient time must be allowed near the end point for the removal of carbon dioxide.

### 8.2. Results

The results of a series of five titrations of this material are given in column 9 of table 1. The precision obtained is comparable to that found with the acids determined by this method.

## 9. Discussion

This investigation shows that the coulometric method is capable of a high degree of precision. In view of the results obtained for benzoic acid and potassium acid phthalate, for which the assay is known, there is substantial evidence that the method is also capable of a high degree of accuracy.

The attainment of precision and accuracy of the order indicated by the data requires careful control over several sources of error. These may be discussed best by a division into errors of procedure and of measurement.

Errors may arise as a result of the pretreatment of the acid to be determined, in its introduction into the cell, and as a result of losses during the titration.

Errors associated with the preparation of the sample are frequently overlooked. In the case of the phthalate, for example, the procedure for drying the sample is critical. It is important that only small quantities be crushed preliminary to drying, in order to release microamounts of entrapped mother liquor from the crystals. Furthermore, the crushing must be done gently, to avoid generation of appreciable heat in localized areas which might result in decomposition of a part of the sample. Also, the sample should be spread out to facilitate the drying process.

To minimize errors associated with the transfer of the sample into the cell, it has been found advantageous to stop the flow of nitrogen during this process. This assures that fine spray will not deposit on the platinum dish to cause an error in weight and also prevents spray losses of material from the cathode compartment.

After the sample has been introduced into the cell, it can be lost only through transfer of solution into the anode compartment, or escape as spray through the nitrogen exit tube. The former is effectively prevented by the agar plug in the connecting tube. The latter is minimized by maintaining only sufficient flow of nitrogen during the titration to guarantee a positive pressure of nitrogen in the cell.

It is very important to remove residual amounts of strong acids from the cell, such as would be introduced during a cleaning operation. This is accomplished by drawing large quantities of hot distilled water through the frits. The effectiveness of the cleaning procedure can be checked when the solution is brought to neutrality at the start of a titration. Prolonged downward drift of *p*H on standing and continuing change of *p*H on rinsing the connecting tube indicate the presence of acid in the frits. Should this situation arise, it is necessary to discard the solution and to rinse the cell further with hot distilled water.

Errors associated with the electrical measurements are easily made much smaller than other errors. Standard resistors, certified to a few parts in a million, are readily obtained, and normal temperature control is all that is required to assure maintenance of their calibration to this accuracy. Calibrated potentiometers permit measurement of the voltage drop, and hence the current, to better than one part in ten thousand. For current measurements of the highest precision, the arrangement used here is preferred, in which the potentiometer measures only the difference between the voltage drop across the resistor and that of a saturated standard cell. This differential method permits measurements with a precision of a few parts in a million.

No difficulties have been experienced in maintaining the current constant to better than one part in a hundred thousand. The galvanometer used to indicate potentiometric balance had a sensitivity of 0.3 *μ*v/mm. Accordingly, a deflection of 3 mm from the null position corresponded to only l*μ*v, or 1 ppm with reference to variation in the current.

It was possible to maintain balance within a few millimeters throughout a determination, provided the variable resistor used for current control had a smooth response. In this connection, a carbon compression resistor, spring-loaded and mounted vertically, is much superior to multiturn potentiometers which have a tendency to become “noisy” with use.

For accurate measurements, it is imperative to insure that the current through the standard resistor is actually that through the cell. While this may seem an obvious condition, the possibilities of extraneous connection to ground (“ground loops”), even though of high resistance, must be considered.

Timing errors should not seriously affect the precision or accuracy of coulometric titrations. For samples of the order of about 10 milliequivalents, approximately 1,000 coulombs are needed, requiring about 5,000 sec at the 200-ma rate of electrolysis. Electric clocks, driven by frequency-controlled power supplies, are inexpensive timing devices which provide measurements reliable to a few parts in a hundred thousand. Perhaps the largest source of error associated with their use is the so-called clutch error involved in starting and stopping, which may amount to a few hundredths of a second per operation. The crystal-controlled electronic time interval meter used here is reliable to a part in a million. Both techniques require that the timing switch operate in close synchronization with the current switch.

Errors arising from the determination of the end point depend on the slope of the *p*H curve in this region, as well as the sensitivity of the *p*H indicating device. In the case of the weak acids used in this investigation (*p*K values, 4.5 to 5.5), the uncertainty of the end point is about 8 millicoulombs, corresponding to errors of less than 10 ppm. For strong acids the end point error is much smaller than this, due to the greater slope of the end point curve, and depends largely on the reliability of the *p*H indication.

Errors in accuracy arise from the deviation of the inflection point in a potentiometric titration from the stoichiometric equivalence point. According to Roller’s equations [9], errors from this source should be less than 0.001 percent. There is some evidence to indicate that the experimental deviations are somewhat greater than the calculated values [6]. However, insufficient work has been done to justify the assignment of corrections to the values reported in this paper.

## 10. Summary

This investigation has shown that coulometric acidimetric titrations can be made with a precision and accuracy equal to or exceeding that obtainable by the most careful conventional chemical methods of analysis. Furthermore, reliable results are less dependent upon the manipulative skill of the analyst than are classical methods. The method has the further advantage of being absolute in the sense that the results are based on electrical standards and no assumptions as to the purity of chemical primary standards are involved. Titrations reliable to a few hundredths of a percent for both strong and weak acids can be readily obtained. The method is equally well-adapted to the titration of bases.

## Figures and Tables

**Table 1:** Summary of results

Determination number	Potassium hydrogen phthalate^a^	Potassium hydrogen phthalate^b^	Benzoic acid^a^	Benzoic acid^b^	Hydrochloric acid^a^ ^c^	Adipic acid^a^ ^d^	Adipic acid^b^ ^e^	Sodium carbonate^a^
	assay	assay	assay	assay	assay	assay	assay	assay
								
	%	%	%	%	%	%	%	*%*
1	99.976	99.979	99.985	99.991	99.982	99.935	99.985	99.909
2	99.980	99.976	99.991	99.993	100.004	99.937	99.987	99.901
3	99.978	99.981	99.989	99.996	100.014	99.937	99.980	99.915
4	99.970	99.976	100.001	99.991	100.003	99.947	99.981	99.910
5	99.974	99.974	99.985	99.986	100.002	99.942	99.989	99.920
6	……………………………………	……………………………………	……………………………………	99.998	99.999	99.955	……………………………………	……………………………………
7	……………………………………	……………………………………	……………………………………	……………………………………	99.990	99.951	……………………………………	……………………………………
8	……………………………………	……………………………………	……………………………………	……………………………………	……………………………………	99.948	……………………………………	……………………………………
								
								
Mean	99.976	99.977	99.990	99.994	99.999	99.945	99.984	99.911
Standard deviation	0.004	0.003	0.006	0.004	0.010	0.007	0.004	0.007

a Timed by electric clock.

b Timed by time interval meter.

c Assay based on calculated acidity by the pressure-distillation method.

d Once recrystallized from melt.

e Twice recrystallized from melt.

## Footnotes

2 Figures in brackets indicate the literature references at the end of this paper.

3 No appreciable error is incurred by assuming the neutral point of the solution to be that of pure water.
